# Imaginary pills and open-label placebos can reduce test anxiety by means of placebo mechanisms

**DOI:** 10.1038/s41598-023-29624-7

**Published:** 2023-02-14

**Authors:** Sarah Buergler, Dilan Sezer, Niels Bagge, Irving Kirsch, Cosima Locher, Claudia Carvalho, Jens Gaab

**Affiliations:** 1 Division of Clinical Psychology and Psychotherapy, Faculty of Psychology, University of Basel, Basel, Switzerland; 2 Institute for Emotion-Focused Therapy, Roskilde, Denmark; 3 Program in Placebo Studies, Beth Israel Deaconess Medical Center and Harvard Medical School, Boston, MA USA; 4 Department of Consultation-Liaison Psychiatry and Psychosomatic Medicine, University Hospital Zurich, University of Zurich, Zurich, Switzerland; 5 Faculty of Health, University of Plymouth, Plymouth, UK; 6 Department of Clinical and Health Psychology, Instituto Superior de Psicologia Aplicada (ISPA), Lisbon, Portugal

**Keywords:** Outcomes research, Placebo effect

## Abstract

Placebos have been shown to be beneficial for various conditions even if administered with full transparency. Hence, so-called open-label placebos (OLPs) offer a new way to harness placebo effects ethically. To take this concept one step further, this study aimed at evaluating placebo effects without the use of a physical placebo, i.e., by imagining taking a pill. Healthy students (*N* = 173) with self-reported test anxiety were either randomized to an imaginary pill (IP; n = 55), an OLP (n = 59) or a control group (CG; n = 59). Both intervention groups were instructed to take two pills daily for three weeks. Primary outcome was test anxiety, secondary outcomes were sleep quality, general well-being and test performance. Groups test anxiety differed at study-endpoint, *F*(2,169) = 11.50, *p* < .001. Test anxiety was lower in the intervention groups compared to the CG, *t*(169) = − 4.44, *p* < .001, *d* = − 0.71. The interventions did not differ significantly, i.e., both were similarly efficacious, *t*(169) = 0.61, *p* = .540, *d* = 0.11. The interaction between group and time in explaining test anxiety was significant, *F*(5,407.93) = 6.13, *p* < .001. OLPs and IPs reduced test anxiety in healthy participants compared to the CG. This finding opens the door for a novel and ethical method to harness placebo effects.

## Introduction

Placebo effects are clinically highly relevant and the need to harness these effects has been voiced^1^. In this regard, open-label placebos (OLPs) administered with full disclosure and transparency can be deemed both ethical and feasible as they avoid the use of deception^2^. Interestingly, meta-analyses show medium sized to large clinically relevant effects of OLPs in patients with various clinical conditions compared to control groups^3,4^. Thus, if placebos also work without deception, it implies that it is not necessarily the pill serving as a symbol for a real medication that triggers these effects. The investigation of underlying mechanisms by eliminating the physical treatment constituent (i.e., the pill itself) can reveal the power of the purely psychological component of a placebo. For this reason, we aimed to evaluate placebo effects without the use of a placebo by having participants imagine taking a pill rather than actually taking one.

The concept of an imaginary pill (IP) was first introduced by De Shazer in 1984 in the context of clinical hypnosis^5^*.* More recently, Niels Bagge, a Danish clinician, independently introduced the same idea without hypnosis^6^. Although seemingly farfetched, recent data supports its plausibility: For instance, pharmacological placebos can be effective even when only possessed, but not applied^7^. Also, psychotherapeutic, non-pharmacological placebos have been shown to be effective^8^ and the idea of triggering placebo effects without a placebo pill is discussed in sports performance^9^, healthcare^10^ and in research on the moderating role of mind-sets^11^. Additionally, a study by Peerdeman et al.^12^ indicated that mental imagery of reduced pain can induce placebo-like expectancy effects on pain. Thus, placebos can also be purely psychological in nature and still produce beneficial effects. With regard to the underlying mechanisms of such psychological placebos, it yet needs to be investigated, whether their efficacy is purely mediated by the meaning that is attributed to these rituals or the expectations of improvement that are being formed as a consequence^13,14^. Despite the elimination of the physical stimulus, it is plausible that an IP relies in principle on the same underlying mechanisms as an OLP. Besides expectation, conditioning could for instance play a role, as even imagining something can activate corresponding brain areas and associated learning mechanisms (e.g.^15^). In addition, placebo mechanisms have also been discussed in relation to the theory of embodied cognition, which states that our experiences are not only consciously stored as memories, but also directly imprinted in our bodies without any cognitive process being involved^16^. Thus, a placebo effect could result from the unconscious internal act of imagining a specific change in body state^16^, which underlines the potential of IPs to harness placebo effects. In conclusion, this study provides a next step towards the quantification of the combined effect of various plausible psychological mechanisms within placebo research, omitting the physical treatment component.

In light of the high prevalence of test anxiety, affecting for example 53% of German freshman medical students^17^, and its negative impact on educational performance^18,19^, this condition is suitable to test the effects of an IP and OLP intervention. Evidence suggests that OLPs can effectively reduce test anxiety in healthy college students^20^ and can have a positive impact on subjective well-being, whereas no improvement of exam performance by the intervention was found^21^. Furthermore, placebo effects in psychopharmacological treatments of anxiety disorders in general^22,23^, social anxiety^24,25^, generalized anxiety disorders^26^ and panic disorders^27,28^ are moderate to large.

In the present study, we set out to test the efficacy of an IP and OLP intervention in reducing test anxiety in a randomized controlled trial with healthy participants. To pursue this research question, we applied a previously used OLP intervention (e.g. in^29,30^) and further developed an IP intervention that was based on knowledge derived from placebo and imagination research to compare them to a control group (CG). We hypothesized that students receiving the OLP and IP intervention would show greater decreases in test anxiety from baseline to study endpoint (shortly before the exam) compared to students in the CG. We further expected students in the intervention groups to show higher general well-being, higher sleep quality and higher test performance than students in the CG.

## Results

### Sample characteristics and study flow

**Figure 1:**
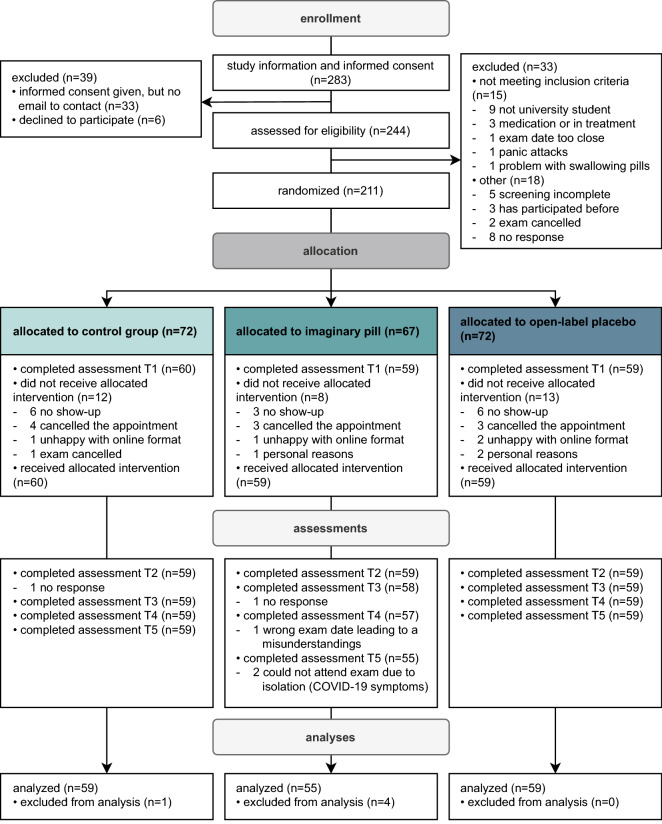
CONSORT diagram. Flow of the study, including reasons for exclusions.

As shown in Fig. 1, of the 283 interested participants, 33 did not provide an e-mail contact and six did not give informed consent. The remaining 244 participants completed the online screening, of which 15 did not fulfill at least one inclusion criteria and 18 were excluded due to other reasons. Hence, 211 participants were randomized, of whom 178 received the intervention and completed the baseline assessment (T1; see Fig. 1 for reasons of exclusion). Five participants were excluded from the analyses as there was missing data (mostly due to nonattendance at exams because of COVID-19). Hence, an *N* of 173 was used for the final analyses (IP = 55, OLP = 59, CG = 59).

Table 1 depicts participants demographic and baseline characteristics. Participants’ age ranged from 18 to 47 years, with a mean of 22.70 (± 4.18) years. The majority were female (85.55%) and undergraduate psychology students (87.86%). The three groups did not significantly differ in any of the demographic characteristics or primary and secondary outcomes at baseline. All outcomes were within the normal range of scores in the anxiety, well-being, and sleep questionnaires, indicating that our sample was healthy displaying an average test anxiety score.

**Table 1:** Demographics and baseline scores of primary and secondary outcomes per group.

	IP	OLP	CG
N (% female)	55 (82%)	59 (90%)	59 (85%)
Age in years, M (SD)	23.20 (4.30)	22.00 (3.48)	22.95 (4.67)
Psychology students, N (%)	49 (89%)	52 (88%)	51 (86%)
Test anxiety (PAF), M (SD)	45.53 (6.81)	48.34 (6.94)	47.36 (6.37)
Sleep quality (PSQI), M (SD)	5.69 (2.83)	6.02 (2.92)	5.85 (3.05)
General well-being (ASS-SYM), M (SD)	45.40 (20.90)	49.03 (22.52)	48.86 (20.76)

*ASS-SYM* Änderungssensitive Symptomliste (general well-being), *CG* control group, *IP* imaginary pill, *M* mean, *OLP* open-label placebo, *PAF* Prüfungsangstfragebogen (test anxiety questionnaire), *PSQI* pittsburgh sleep quality index, *SD* standard deviation.

### Primary and secondary outcomes at study endpoint (T4)

**Figure 2:**
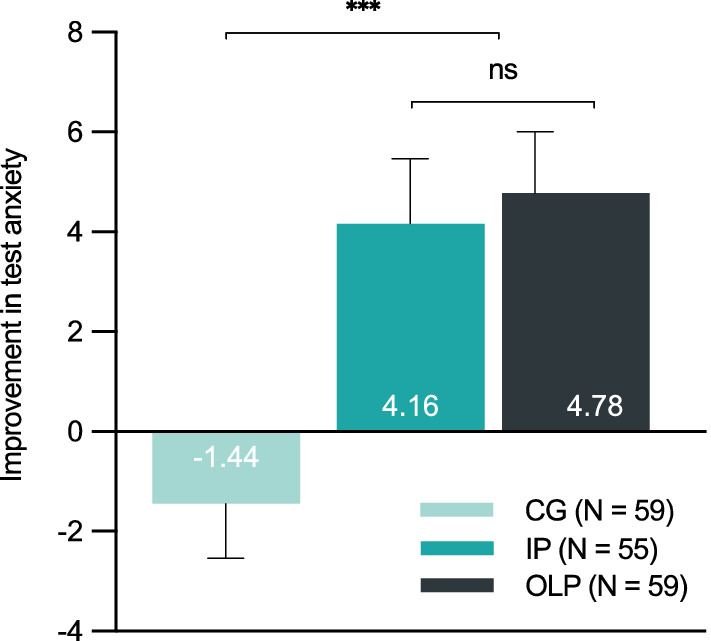
Mean improvement in test anxiety (PAF: test anxiety questionnaire) from baseline (T1) to endpoint (T4) per group. Results indicate a significant improvement for the OLP and IP group compared to the CG. *Note.* *CG* control group, *IP*  imaginary pill, *ns* = not significant, *OLP* open-label placebo, ***p < .001. Error bars represent standard error of the mean.

Figure 2 shows mean improvement from baseline (T1) to endpoint (T4) per group on the primary outcome. Table 2 depicts all primary and secondary outcomes for all groups and assessments.

**Table 2:** Mean values for primary and secondary outcomes per group at all assessed timepoints.

		T1	T2	T3	T4	T5
	Group (*N*)	M (SD)				
PAF	IP (55)	48.85 (9.20)	45.25 (8.26)	43.84 (8.86)	44.69 (9.72)	39.85 (10.18)
	OLP (59)	52.36 (9.18)	49.31 (7.81)	46.70 (8.49)	47.58 (9.39)	41.64 (10.60)
	CG (59)	50.66 (7.37)	52.08 (7.70)	51.47 (8.23)	52.10 (9.56)	45.78 (9.97)
ASS-SYM	IP (55)	45.40 (20.90)	42.55 (22.57)	37.29 (21.9)3	39.25 (23.04)	
	OLP (59)	49.03 (22.52)	46.56 (19.48)	44.58 (19.96)	42.85 (21.99)	
	CG (59)	48.86 (20.76)	52.85 (24.36)	51.81 (22.02)	55.80 (28.58)	
PSQI	IP (55)	5.69 (2.83)	5.69 (2.48)	5.49 (2.46)	5.54 (2.71)	
	OLP (59)	6.02 (2.92)	5.49 (2.52)	5.88 (3.08)	5.86 (2.82)	
	CG (59)	5.85 (3.05)	6.36 (2.94)	6.36 (2.90)	6.22 (3.00)	

*ASS-SYM* Änderungssensitive Symptomliste (general well-being), *CG* control group, *IP* imaginary pill, *M* mean, *OLP* open-label placebo, *PAF* Prüfungsangstfragebogen (test anxiety questionnaire), *PSQI* pittsburgh sleep quality index, *SD* standard deviation.

The overall analyses of covariance (ANCOVA) showed that groups significantly differed in test anxiety at study endpoint T4, *F*(2, 169) = 11.50, *p* < .001. Planned contrasts indicated that the mean changes in test anxiety were significantly greater in the intervention groups (OLP/IP) compared to the CG at study endpoint T4, *t*(169) = − 4.44, *p* < .001, *d* = − 0.71. However, changes in test anxiety did not differ between the two intervention groups, *t*(169) = 0.61, *p* = .540, *d* = 0.11. These results held true for all subscales of the test anxiety questionnaire (see supplementary Table S1).

Regarding secondary outcomes, the groups differed significantly in terms of general well-being at study endpoint T4, *F*(2, 169) = 9.37, *p* < .001 with the same pattern across all subscales. Changes in general well-being were significantly greater in the intervention groups (OLP/IP) compared to the CG at study endpoint T4, *t*(169) = − 3.98, *p* < .001, *d* = − 0.64, but did not differ between the two intervention groups, *t*(169) = 0.38, *p* = .707, *d* = 0.07.

No significant between-group effect was found for total sleep quality, *F*(2, 169) = 0.902, *p* = .408, or in any of its component subscales. Nevertheless, although the overall between-group effect on the subjective sleep quality at study endpoint failed to reach statistical significance, *F*(2, 169) = 2.73, *p* = .068, contrasts indicated that both intervention groups showed better subjective sleep quality compared to the CG, *t*(169) = − 2.40, *p* = .017, *d* = − 0.39, with no significant difference between the intervention groups, *t*(169) = 0.06, *p* = .952, *d* = 0.01.

**Figure 3:**
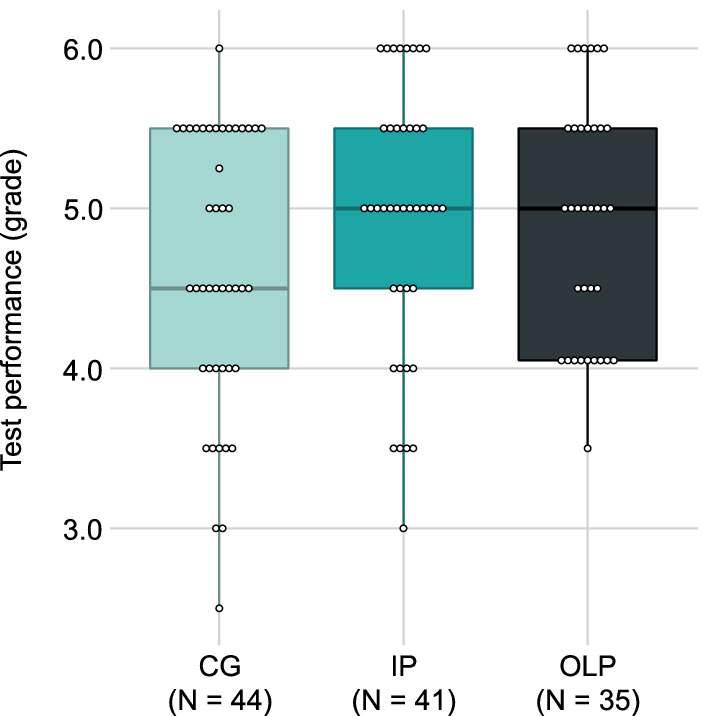
Boxplot showing continuous grades of the participants per group. Every dot represents a participants’ grade with higher grades being better (ranging from 1.0 to 6.0). *Note.* Median is represented by the bold line within the box and upper/lower quartiles mark the end of the box. *CG* control group, *IP* imaginary pill, *OLP* open-label placebo.

With respect to the test performance, 120 participants had a continuous test score (IP = 41, OLP = 35, CG = 44). Mean grade was 4.82 (± 0.83) ranging from 2.5 to 6.0 (IP = 4.94 ± 0.83, OLP = 4.92 ± 0.76, CG = 4.62 ± 0.87). Figure 3 depicts the participants grades per group. The overall ANOVA showed no significant group effect on test score, *F*(2, 117) = 1.98, *p* = .143. The contrasts, however, indicated that the intervention groups (OLP/IP) had higher test scores compared to the CG, *t*(117) = 1.98, *p* = .050, *d* = 0.38, whereas the intervention groups did not differ, *t*(117) = − 0.12, *p* = .908, *d* = − 0.03. Binary test scores (pass/fail) revealed that 155 (89.60%) of all participants passed the exam (IP = 87.27%, OLP = 96.61%, CG = 84.75%).

### Primary and secondary outcomes over time (T1–T4)

**Figure 4:**
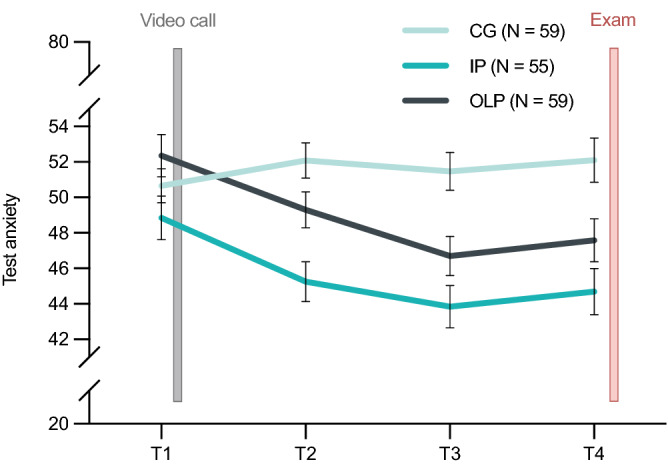
Course of test anxiety over time. Mean test anxiety per group from baseline (T1) through midpoints (T2, T3) to study endpoint (T4). *Note.* Error bars represent standard error of the mean. *CG* control group, *IP* imaginary pill, *OLP*  open-label placebo.

Figure 4 shows the course of test anxiety outcomes over time. There was a statistically significant interaction between group and time (T1–T4) for test anxiety, *F*(5, 407.93) = 6.13, *p* < .001. Bonferroni adjusted post-hoc p-values showed that the simple main effect of group was significant after one week (T2; *p*_adj_ < .001), two weeks (T3; *p*_adj_ < .001) and three weeks (T4; *p*_adj_ < .001) after randomization, but not at baseline (T1; *p*_adj_ = .098). Furthermore, there was also a statistically significant effect of time on test anxiety scores for the IP (*p*_adj_ < .001) and OLP (*p*_adj_ < .001) group, but not for the CG (*p*_adj_ = .318).

Regarding the secondary outcomes, a statistically significant interaction was found between group and time (T1–T4) in general well-being, *F*(5, 422.71) = 3.58, *p* = .004. Considering the Bonferroni adjusted p-values, the simple main effect of group was significant at T3 (*p*_adj_ = .004) and T4 (*p*_adj_ = .004), but not at T1 (*p*_adj_ = .598) and T2 (*p*_adj_ = .061). Also, the effect of time was significant with an increase of general well-being in the IP (*p*_adj_ < .01), but not in the OLP (*p*_adj_ = .191) group, whereas general well-being in the CG showed a trend to decrease (*p*_adj_ = .071). There was no significant interaction between group and time on sleep quality scores, *F*(5, 443.85) = 0.90, *p* = .485.

### Rating of test anxiety at follow up, opinion on treatment idea, side-effects and adherence

Regarding the retrospective evaluation of the test situation (T5), the overall ANCOVA showed a significant overall effect of group, *F*(2, 169) = 5.89, *p* = .003. Planned contrasts indicated that mean retrospective test anxiety scores were rated significantly lower in the intervention groups (OLP/IP) compared to the CG at T5, *t*(169), = − 3.29, *p* = .001, *d* = − 0.53. However, retrospective test anxiety scores did not differ between the two intervention groups, *t*(169) = 0.10, *p* = .918, *d* = 0.02.

Table 3 provides an overview of the evaluation of the idea (positive, negative, neutral) towards the two interventions in the context of the open questions. The two independent raters had concordant judgments for 91.2% of the answers. A third rater was included for the remaining 8.8%.

**Table 3:** Ratings of the open-ended questions. Judgement of the idea regarding the respective interventions.

	OLP (*N* = 59) *N* (%)	IP (*N* = 55) *N* (%)
Positive	39 (66.1%)	38 (69.1%)
Negative	8 (13.6%)	10 (18.2%)
Neutral	12 (20.3%)	7 (12.7%)

*IP* imaginary pill, *OLP* open-label placebo.

No negative side-effects were reported, other than in the IP group, in which three subjects mentioned additional effects immediately after pill intake (i.e., dry mouth, goose bumps, warmth radiating from the abdomen). These effects were suggested during the pill intake in the study contact and were part of the IP response to demonstrate the effect of the pill (see supplementary material).

Regarding adherence, one participant (1.7%) in the OLP group and five participants (9.1%) in the IP group reported less than 80% adherence (i.e., forgot 9 or more pills).

### Influence of study contact duration, treatment provider and moderation of treatment expectancy on primary outcome

Study contact duration was significantly associated with changes in test anxiety from T1 to T4, *F*(1, 168) = 5.84, *p* = .017. However, when including treatment group as an additional factor in the model, contact duration was no longer significant, *F*(1, 166) = 0.01, *p* = .942 and group remained significant, *F*(2, 166) = 8.00, *p* < .001. Treatment provider was not associated with changes in test anxiety, *F*(1, 171) = 0.80, *p* = .373.

Mean expectancy of relief across the 20 items of the test anxiety questionnaire was significantly different across the three groups,* F*(2, 170) = 14.86, *p* < .001. Participants receiving an intervention (IP/OLP) expected less symptoms compared to the CG, *t*(169) = − 5.76, *p* < .001, *d* = − 0.92, whereas scores of the two intervention groups were comparable, *t*(169) = 1.47, *p* = .144, *d* = 0.28. Mean expectancy of relief measures significantly correlated with endpoint test anxiety (T4; *r* = 0.56, *p* < .001). When including expectancy of relief as an additional covariate into the overall model, expectancy of relief was significantly associated with test anxiety, *F*(1, 168) = 21.14, *p* < .001, but treatment group remained significant, *F*(2, 168) = 12.87, *p* < .001.

## Discussion

The present randomized controlled trial tested the effects of an IP against an OLP intervention and a CG on test anxiety in healthy students. We found that both IP and OLP significantly reduced test anxiety compared to the CG with a moderate-to-large effect size (d = − 0.71). These findings were comparable across all subscales of the test anxiety questionnaire (i.e., worry, emotionality, interference and lack of confidence). Interestingly, the beneficial effect was apparent over the course of the three weeks, starting after only one week of intervention*.* While study contact duration and treatment provider did not appear to be critical for changes in test anxiety, the observed effects were associated with treatment expectancy as this measure positively correlated with changes in test anxiety (r = 0.56). The retrospective assessment of the exam situation (follow-up T5) supports the superiority of the two interventions over the CG, as it indicated less retrospectively perceived anxiety during the exam situation*.* Consistent with the effects on our primary outcome, general well-being was significantly augmented in both intervention groups compared to the CG with a moderate to large effect (d = − 0.64). Overall sleep quality, however, was not affected by the intervention, i.e., all three groups showed comparable sleep quality during the three weeks. Test performances (i.e., continuous grades) were significantly better in the intervention groups compared to the CG with a small effect (d = 0.38). Overall, OLP and IP showed comparable results on all assessed outcomes. These findings question the necessity of the pill to produce positive treatment effects.

The effect sizes of the two interventions in the present study are slightly higher compared to a previous OLP trial testing openly prescribed placebos in test anxiety against no treatment with a between group effect size of d = 0.54^20^, whereby test anxiety scores in both studied populations indicate average, non-clinical test anxiety^31^. The remarkable and rapid decreases in test anxiety in the intervention groups of the present study are noteworthy. The observed effect is comparable to the moderate-to-large effect (g = − 0.76) of a meta-analysis on various psychological interventions for test-anxious university students (i.e., psychological, study skill training, and/or combined intervention packages) against control conditions^32^.

Extended or different placebo paradigms such as IPs aid to understand the mechanisms of OLP by systematically manipulating the treatment setting and application. As OLP and IP groups showed comparable results in all outcomes, the necessity of a physical placebo to produce positive treatment effects is called into question. Psychological components, for their part, may be sufficient on their own to exploit placebo effects which is supported by studies showing that triggering placebo effects without a physical treatment component is possible^8,11,33^. Research on placebo-like expectancy effects in pain analgesia is consistent with this: Peerdeman et al. (2017) showed less experienced pain in participants receiving instructions to vividly imagine a warm and impermeable glove preventing pain from cold before a cold pressor test, compared to a control imagery group instructed to imagine their hand without any reference to pain or cold water. This effect was mediated by expected pain^12^. Along these lines, expectancy of relief was also significantly associated with test anxiety in our study. However, the treatment group remained significant even after expectancy was included in our linear model, implying that not only expectancy but also other factors must account for the group-specific improvement in test anxiety. The effects can, for example, be discussed in the context of the embodied cognition theory, which states that a placebo effect can result unconsciously from embodied experiences by an internal act of imagining a particular state change in the body^16^. Similarly, conditioning effects may have played a role in our study, as even imagining something can activate corresponding brain areas (e.g.^15^). The Western cultural understanding of a pill underpins this line of reasoning as a pill in itself has a therapeutic meaning—learning from an early age to associate the pill and its effects, whereas no physical pill is required to trigger positive processes. Notably, mental imagery relies on similar neural processes to those of actual perception^34,35^. The ability to generate internal representations that retain the essential features of a perceptual experience suggests that mental imagery may have similar effects to actual experiences^12^. Consistent with the response expectancy theory^14^ the findings of this study extend previous research on the mechanisms of placebo effects by showing that placebo effects on test anxiety can be induced not only by a physical cue, but also by imagining a pill and its effects. Overall, it can be suggested that OLP and IP may rely on the same underlying mechanisms (e.g., expectations, conditioning, embodied cognition), whereas these mechanisms can be triggered even in the absence of a physical pill.

Due to the COVID-19 pandemic, the study contact took place by means of a virtual clinical encounter. The present study is not the first to provide the OLP treatment remotely: Kube and colleagues^36^, however, failed to replicate previous findings of OLP effects on allergic rhinitis^37,38^. They concluded that remote OLP provision is feasible, yet their effectiveness might be lower, as a physical encounter between patient and provider might be a prerequisite for OLPs to be effective^36^. However, our findings demonstrate that providing OLP and IP remotely is not only feasible but can also yield significant effects. A potential reason for the better effects in this online intervention compared to Kube et al. might be the younger sample (22.7 vs. 31.1 years) consisting only of students who may be more accustomed to online interactions. Whether the effects would be different with physical contact remains unclear and should be tested in a follow-up study.

This is the first study to conceptually extend ethically feasible placebo treatments by testing an IP intervention for test anxiety, taking OLP research a step further. It moreover corroborates important findings on OLP efficacy in a remote setting on a large sample. A manual including a five-step procedure was developed by our team to implement the IP intervention (see supplementary material). Manualized instructions used in the study further allowed for the control of many incidental factors to make accurate inferences about the interventions tested. Weekly assessments of primary and secondary outcomes moreover enabled observation of placebo effects over time. Also, there were less than 3% participants with missing data and reported nonadherence was low, especially in the OLP group.

Nevertheless, several aspects of this study need to be considered: Due to sample restriction and recruitment locations and routes, a largely female, young, academic sample resulted, limiting generalizability of the findings. Also, most outcome measures were self-reported and rather subjective than objective, raising questions of report and social desirability bias. Disappointment effects may have further played a role in the CG as they were not offered future treatment. In fact, 52.5% reported to be disappointed due to being allocated to the CG. However, given that test anxiety can be assumed to increase as the exam approaches^39^, but the CG showed stable scores over time, it seems that despite disappointment this group also benefited from taking part in the study. In addition, adherence was self-reported, so we had no option to verify the reported values, eliminating the influence of social desirability bias. Further, because of planning reasons, a short time gap between study contact and start of the intervention occurred in some participants. However, this gap was kept to a minimum. Also, the conduct of the present study coincided with the start of the COVID-19 pandemic, which necessitated meeting with participants virtually. Due to the remote setting, the participants in the OLP group received the envelope with the placebo pills in advance: although not knowing about the contents of the envelope and being instructed not to open the envelope until the study contact occurred, we had no way of controlling this behavior. Nevertheless, positive effects of interventions could be observed and implementation remotely was feasible. Considering this, we assessed changes in test anxieties due to the pandemic-related circumstances which were almost evenly distributed across participants—with some reporting unchanged (33.5%), higher (34.1%) or less (32.4%) anxiety. Comparisons of within changes of participants should, however, control for these complicated circumstances. Future investigations should test OLP and IP with physical contact and no pandemic-related restrictions.

The present study is the first to conceptually expand on previous OLP studies by eliminating the physical pill as a treatment component and testing an IP intervention. Results indicate a moderate-to-large effect of both interventions on test anxiety and general well-being in a large cohort of 173 healthy students. These findings demonstrate that placebo effects can be harnessed without the use of a physical pill. The IP intervention could thus serve as a stand-alone or adjunct treatment to maximize and boost placebo effects in clinical practice, as indicated by the ethical principle of “beneficence”^1,40,41^. As an ethical, cost-effective, easily applicable and fully patient-centered method, the IP intervention has potential and should be tested in other settings, conditions and populations.

## Methods

### Experimental design

Between March 2020 and July 2021, we conducted an online randomized controlled, parallel group trial at the Division of Clinical Psychology and Psychotherapy at the Faculty of Psychology, University of Basel, Switzerland, in order to test the effects of an IP and OLP intervention compared to a CG in healthy students with test anxiety. Written informed consent was obtained from each participant before participation in the study. The Ethics Committee of the Faculty of Psychology, University of Basel approved the design and informed consent of the study. This study was carried out in accordance with the protocol and principles enunciated in the current version of the Declaration of Helsinki. The study was registered at ClinicalTrials.gov: NCT04250571 (31/01/2020).

### Study population

Participants were recruited via web- and print-based advertisement (title: “Efficacy study of two treatment methods for test anxiety”) and registered online for the study. Potential enrollees had to be students of the University of Basel aged between 18 and 65 years. To meet inclusion criteria, participants had to have an exam at the end of the semester, have self-reported test anxiety, being healthy by self-report (i.e., no known current or chronic primary pain disorders or psychiatric disorders) and be sufficiently proficient in German. Exclusion criteria were use of medications (psychoactive or narcotic), being in psychological or psychiatric treatment, taking psychotropic drugs, being a master student in Psychology (due to prior knowledge about placebo mechanisms), allergy to one of the ingredients of the placebo pills (see supplementary material), and problems swallowing pills. All participants were reimbursed either financially or with credit points.

### Study procedure

**Figure 5:**
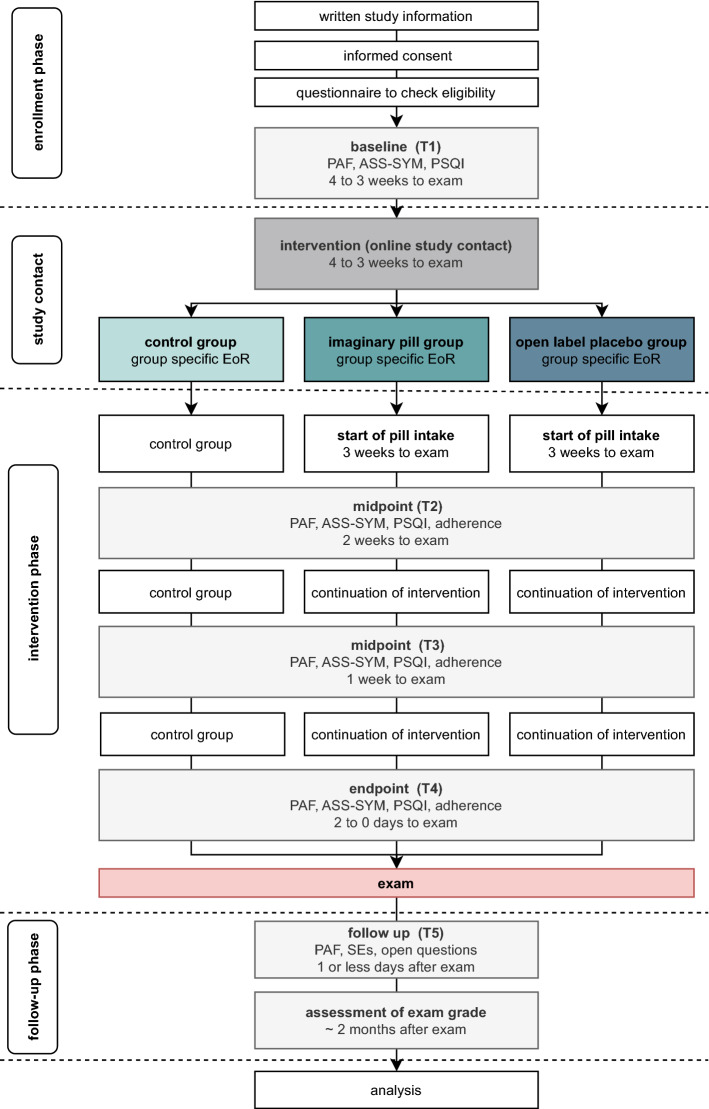
Procedure of the study. *Note:* *ASS-SYM* Änderungssensitive Symptomliste (general well-being), *EoR* expectancy of test anxiety relief, *PAF* Prüfungsangstfragebogen (test anxiety questionnaire), *PSQI* pittsburgh sleep quality index, *SEs* side-effects, *SDD* sociodemographic data.

The study procedure is depicted in Fig. 5. Interested participants were directed to an online survey page providing information about the nature and purpose of the study. Upon providing online informed consent, participants were checked online for inclusion and exclusion criteria. Eligible participants were randomly assigned to one of three study group. Baseline assessments of primary and secondary outcomes were completed online two or less days before the study contact (T1). The study contact, in which participants received one of three interventions (four to three weeks before the exam), was held online via the standard video call software of the University of Basel, zoom (https://zoom.us/), the use of which was approved by the ethics committee. Expectancy of relief was assessed immediately after the study contact. Study contacts were distributed over the time period of four to three weeks before the exam for resource management reasons (number of treatment provider). However, treatment started exactly three weeks before the exam as indicated by the receipt of a reminder e-mail in both intervention groups, i.e., the treatment duration was the same. Again, two weeks (T2), one week (T3) and two or less days before the exam (T4) all three groups were asked to complete online assessments of primary and secondary outcomes, as well as to answer one question regarding their intervention adherence. After the exam, there was a final online assessment (T5) to evaluate retrospective experiences of the exam situation, to assess side-effects during the treatment period and to answer open questions respective to the group (e.g., possible feelings of disappointment to be assigned to the CG, see supplementary material). Finally, all participants were asked about their examination grade (approximately two months after the exam).

### Study arms

In total, there were three study arms, i.e., CG, OLP, and IP.

Participants allocated to the IP group did not take a physical pill, but imagined taking a pill along with verbal suggestions from the treatment provider during the study contact. Hence, the idea of IPs has resemblance to the clinical application of hypnosis^42^. Participants in this group received a procedure in accordance with the technique by de Shazer^5^ and a structure proposed by Niels Bagge^6^. Detailed formulation and translation to German was performed by the local study team (SB, DS, CL, JG). The instruction consisted of a procedure including five steps: (1) identifying the persons’ problem and the desired state, (2) building trust in the treatment, (3) constructing a personally meaningful pill, (4) taking the IP, (5) suggestions for self-administration in real life and building adherence (see supplementary material). Importantly, step 2 consisted of teaching participants about findings of (open-label) placebo and imagination research. At the end of the intervention, participants in the IP group had to describe their individual elaborated pill (size, shape, pill kind, color, packaging) and its effects in an interactive document. They sent the completed document back to the treatment provider and were able to print it out for their own use. Participants were asked not to take any physical aids, such as a candy, to facilitate their imagination, ensuring that the groups remained distinguishable in their specific ingredients. Participants were instructed to take two IPs a day for three weeks until the exam takes place and received daily e-mail reminders during that period to remember their IP intake.

In the OLP group participants obtained the information that they were receiving inert blue pills (i.e. "P-Dragees" blau Lichtenstein manufactured by Zentiva Pharma GmbH) and were given a treatment rationale in accordance with previous OLP studies (e.g.,^29,30^; see supplementary material), that encompassed four discussion points. In order to keep the OLP rationale similar to the one of the IP, a brief introduction was added at the beginning of the intervention, elaborating on what comprises the persons’ problem (how do symptoms express themselves; how does the person wish to feel). Hence, the rationale was structured as follows: (1) identifying the OLP-sensitive problem, (2) deceptive as well as OLPs are efficacious, (3) one mechanism of placebo is conditioning, (4) an open attitude towards the treatment can be helpful but is not necessary for its effect, (5) taking the pill faithfully is important. Participants were instructed to take two placebo pills a day for three weeks until the exam takes place. The placebo pills were sent in an envelope to participants via postal mail prior to the online study contact or if participants did not wish to disclose their postal addresses, they were given the option of a personal handover by a member of the study team. Participants did not know about the content of the envelope and were instructed to not open the envelope until the study contact takes place. Daily e-mails were also sent to this group as a reminder to take the placebo pills.

In order to control for factors not considered characteristic for the intervention, the CG was fashioned according to the intervention groups (i.e., characteristic components were the pill intake and intervention-specific rationales^43^). Participants were (1) reminded of the importance of this group, (2) asked about the nature of their exam, (3) about their problem (i.e., test anxiety) and the wished-for state, (4) and about learning strategies. The design of this group attempted to keep interaction time comparable and to account for the structural equivalence between the CG and intervention groups, e.g., by allowing the CG to talk about the problem (i.e., test anxiety) to enable a "fair" comparison of groups^44^. Despite the interventional nature of this study arm, no advice or problem-solving task was given (see supplementary material).

Study contacts on zoom were carried out by five female treatment providers. Although not all treatment providers had the same number of study contact appointments, the proportion of participants per group were evenly distributed among them. Average duration of interventions was 31 minutes (IP = 44 min, OLP = 29 min, CG = 20 min).

### Randomization and blinding

A random allocation sequence was created by SB using the built-in random number generator in Microsoft Excel for Mac, version 16.53. Participants were enrolled in the pre-generated list in order of their study registration and assigned by master students to interventions accordingly. All participants were informed about their assigned group at the study contact via zoom. Due to the study design, the providers were unblinded to the treatment they were administering. However, the encounter was kept constant in all groups through a standardized protocol. Also, except for the study contact on zoom, all communication was via e-mail contact (e.g., sending links for online assessments), using the same e-mail templates for all three groups to ensure the same type of interaction.

### Outcome measures

The primary outcome was test anxiety measured by means of the “Prüfungs-Angst Fragebogen” (PAF; English: “test anxiety questionnaire”^31^). The questionnaire consists of 20 items with four subscales (worry, emotionality, interference, lack of confidence) with scores ranging from 20 to 80 points. Each item is rated on a 4-point Likert scale (*1—almost never* to *4—almost always*). Secondary outcomes were sleep quality and general well-being. Sleep quality was assessed by means of the “Pittsburgh Sleep Quality Index” (PSQI^45,46^). The PSQI is an 18 item self-rating questionnaire forming 7 subscales. To fit our time frame, we adjusted the time interval to the last week (7 days). To assess general well-being the ASS-SYM symptom list was used (Änderungssensitive Symptomliste^47^). This list is composed of 48 items and 6 subscales. Lower scores indicate less symptoms (i.e., higher general well-being). All measures were assessed four to three weeks prior to the exam (T1; baseline assessment), two and one week prior the exam (T2–T3; midpoint assessments) and two or less days prior the exam (T4; endpoint assessment).

Test performance of each participant was collected as another secondary outcome (approximately two months after the exam). Students received as a test performance either a continuous grade, ranging from a minimum of 1 (very poor) to a maximum of 6 (very good) in the Swiss grading system, or a binary test score (pass or fail), where a grade greater than or equal to 4 (sufficient) is considered a pass. Other outcomes of interests included sociodemographic data (SDD) assessed at T1. Immediately after the intervention, expectancy of test anxiety relief^48^ according to the received intervention was assessed using an ad-hoc constructed questionnaire with each item of the primary outcome on a numeric rating scale from 1 to 4 (e.g., based on the intervention you have received, how strong would you expect the following symptoms to be present before your next exam on a scale from 1—*almost never* to 4—*almost always*) as e.g. used in^49^. Furthermore, within the intervention groups, adherence was assessed weekly with a single item asking for how often someone forgot the actual or imagined pill intake in the last week. In total, each participant assigned to one of the two intervention groups had to take 42 pills (i.e., 2 pills × 21 days). A sum score was computed to determine overall adherence. Adherence was defined as > 80% (i.e., 9 or more missed pills). Additional variables were collected on the same day or at most one day after the exam (T5; follow up assessment) including retrospective experience of the exam using the test anxiety questionnaire (i.e., the wording of the introduction was changed as follows: please read through each statement and choose from the four answers 1—*almost never* to 4—*almost always* the one that indicates best how you were feeling during the exam), side-effects (i.e., (1) did you experience side effects, (2) if yes, give a description, (3) how severe were they from 0—*none* to 100—*very severe*, (4) when was the onset, (5) how long did the they last, (6) was there a connection with participation in our study?) and open questions respective to group allocation for example about the idea of intervention (i.e., what do you think about the idea of taking placebo/imaginary pills?; see supplementary material for all open-ended questions). All outcome variables were assessed by means of online surveys using Limesurvey (limesurvey.org).

### Statistical analyses

Statistical analyses were carried out using the open-source software environment RStudio. For all analyses, significance level was set at *α* = 5%. Using a conservative power analysis on the basis of an *F*-Test and an ANCOVA for three groups, we calculated that a total sample size of *N* = 206 for a power of 0.9 and a total sample size of *N* = 158 for a power of 0.8 would be necessary to detect a medium effect size of *f* = 0.25 (i.e., *d *= 0.5) with an alpha-level of 0.05, using the statistical software G*Power. On this basis we decided on a total sample size of a minimum of 165 participants. Considering dropouts (e.g., due to increased nonattendance because of the COVID-19 pandemic), we planned to include and randomize slightly more than 55 per group (*N* ~ 60). Cohen’s *d* was used to assess the size of effects.

Initially planned multiple imputation was not conducted, as there were less than 3% participants with missing data and the missingness appeared to be completely random (e.g., due to nonattendance at exams because of COVID-19). The five participants with missing data were thus not considered for analyses (see Fig. 1 for reasons for exclusion).

To assess differences in changes from baseline (T1) to endpoint (T4; primary analyses) and follow-up (T5) in test anxiety across the three groups, two separate omnibus tests (ANCOVA) using treatment group as the independent factor and baseline (T1) as covariate to control for baseline differences^50^ were computed to test for overall effects. Orthogonal contrasts were computed to evaluate intergroup differences in the change from baseline (T1) to study endpoint (T4). The contrasts were: CG < IP + OLP and IP < OLP. To evaluate changes over time, we conducted a two-way mixed analysis of variance (ANOVA) using group as between-subject factor and time (T1-T4) as within-subject factor. Bonferroni adjustments accounted for multiple testing within post-hoc tests.

To analyze test performance across groups, we followed a two-step approach as there were continuous (grades) as well as binary (pass/fail) test scores. First, we performed an analysis only with participants having a continuous test score (1–6) using an ANOVA to test for overall effects and above-mentioned contrasts. Second, all continuous variables were transformed into a binary test score (pass ≥ 4; fail < 4) and reported as percentages of passing.

In order to investigate differences in treatment expectancy of relief across groups, an overall ANOVA was performed using the expectancy scores as outcome and group as between-subject factor. A priori contrasts were then used to explore differences across groups. Furthermore, we calculated correlations in order to investigate possible relationships between treatment expectancy of relief and test anxiety^51^ and computed a linear model with test anxiety from T1 to T4 as dependent factor and expectancy of relief as independent factor to investigate their impact on the effects.

To investigate the influence of study contact duration and treatment provider on test anxiety we used a linear model with the corresponding variable as independent factor and changes in test anxiety from T1 to T4 as dependent variable. To analyze the open-ended questions about attitudes toward the idea about the two interventions, two independent raters rated each statement as "positive," "negative," or "neutral". When ratings differed, a consensus was reached by a third rater.

## Supplementary Information

Supplementary Information.

## Data Availability

Access to data from this study may be obtained by contacting the corresponding author.
