# Supplementary material for: Imaginary pills and open-label placebos can reduce test anxiety by means of placebo mechanisms
Source: Sci Rep. 2023 Feb 14;13:2624. doi: 10.1038/s41598-023-29624-7 (PMC9926426; doi:10.1038/s41598-023-29624-7)
Supplement: Supplementary file 1 — Supplementary Information. [file 41598_2023_29624_MOESM1_ESM.pdf]

Supplementary Materials for

Imaginary pills and open-label placebos can reduce test anxiety by means of placebo mechanisms

Sarah Buergler, Dilan Sezer, Niels Bagge, Irving Kirsch, Cosima Locher, Claudia Carvalho, & Jens Gaab

This file includes:

Supplementary Text

Supplementary Table

Supplementary Text

Ingredients of placebo pills

Lactose-Monohydrat, Magnesiumstearat, mikrokristalline Cellulose, hochdisperses Siliciumdioxid, weisser Ton, Macrogolglycerolhydroxystearat, Arabisches Gummi, Montanglycolwachs, Povidon, Talkum, Titandioxid, Patentblau-V-Aluminiumsalz, Calciumcarbonat, Sucrose, Glukosesirup, Maisstärke, Macrogol 6000

Rationales

Imaginary Pill (IP) rationale (translated from German in English)	Open-label Placebo (OLP) rationale (translated from German in English)	Control Group (CG) rationale (translated from German in English)
**1. Identifying the IP-sensitive problem and the desired state**<br>Before I explain the concept of the open administration of placebos and how we use these placebo effects with the imaginary pill intervention, I would like to know more about your test anxiety and your preparation stress. Could you use a previous exam situation to describe what the symptoms feel like? I ask you now to think back to that bad exam/situation. Can you now describe to me, based on this previous exam situation, what the symptoms of your exam anxiety felt like? What are the sensations in your body? What are your thoughts and emotions when you experience this anxiety? Now, thinking of the upcoming exam, if you had to specify how strong these symptoms are from 0-10 (not at all – very strong) in this moment, what would you say?<br>And now can you describe how you would like to feel in your exam phase/during the exam? Can you describe to me a specific situation where you feel this? What are your feelings in this situation?	**1. Identifying the OLP-sensitive problem**<br>Before I explain the concept of the open administration of placebos and how we use these placebo effects with the imaginary pill intervention, I would like to know more about your test anxiety and your preparation stress. Could you use a previous exam situation to describe what the symptoms feel like? I ask you now to think back to that bad exam/situation. Can you now describe to me, based on this previous exam situation, what the symptoms of your exam anxiety felt like? What are the sensations in your body? What are your thoughts and emotions when you experience this anxiety? Now, thinking of the upcoming exam, if you had to specify how strong these symptoms are from 0-10 (not at all – very strong) in this moment, what would you say?<br>And now can you describe how you would like to feel in your exam phase/during the exam? Can you describe to me a specific situation where you feel this? What are your feelings in this situation?	**1. Explaining importance of group**<br>As you already know from the study information, we randomly assign all study participants to one of the three study groups. You have been assigned to the control group, which means you will not receive a treatment. This group and your participation is very important for our study. Only through the control group can we see how symptoms naturally behave when you do not receive a treatment. So, we are also asking you to fill out all the online surveys accordingly. You will still receive weekly surveys.

What are your thoughts? What physical sensations do you have in this situation? Try to put yourself in this positive state.<br>[Check to see if the person really knows how they want to feel. Get as precise as possible].	What are your thoughts? What physical sensations do you have in this situation?<br>Ok, what you say is all very understandable and I hope we can help you with our intervention to reach the positive state you just described. Is it okay if I now explain to you the concept of open placebos?	
**2. Building trust in the treatment**<br>Is it ok if I now explain the concept of open-label placebos and how you can use this placebo effect with the imaginary pill for your goal – the positive experience, we've just talked about? OK, we know from clinical research that placebos have significant effects on pain, depression and anxiety and that these effects can even be demonstrated in changes in brain activity and the release of neurotransmitters. As mentioned earlier, scientists previously assumed that placebo pills can only help if they are given covertly, i.e. with deception. Now, however, more recent studies suggest that this is not the case. This means that placebos can work even if the patient knows that it is a placebo. We are incorporating this approach in our study. Many double-blind randomized studies show that the placebo effect is very effective for many complaints. This means that placebos can relieve pain, cramps and gastrointestinal complaints, among other problems, and also have a very positive effect on mood. Especially for chronic back pain and irritable bowel syndrome, the open-label placebo treatment has been shown to be very effective, even in patients where nothing else has worked. Here at the division, a study has already been carried out in which a placebo cream was	**2. Deceptive and OLPs are effective**<br>We know from clinical research that placebos have significant effects on pain, depression and anxiety and that these effects can even be demonstrated in changes in brain activity and the release of neurotransmitters. As mentioned earlier, scientists previously assumed that placebo pills can only help if they are given covertly, i.e. with deception. Now, however, more recent studies suggest that this is not the case. This means that placebos can work even if the patient knows that it is a placebo. We are incorporating this approach in our study. Many double-blind randomized studies show that the placebo effect is very effective for many complaints. This means that placebos can relieve pain, cramps and gastrointestinal complaints, among other problems, and also have a very positive effect on mood. Especially for chronic back pain and irritable bowel syndrome, the open-label placebo treatment has been shown to be very effective, even in patients where nothing else has worked. Here at the division, a study has already been carried out in which a placebo cream was used for the treatment of heat-induced pain. And there too we found large placebo effects. A positive placebo effect has also been shown for test anxiety. This has been shown recently by a	**2. Nature of exam**<br>In your case, we would be interested in how the exam stress and anxiety manifests itself and what your general learning strategies are. I will possibly make some notes on this. Before we get to your exam anxiety itself, I'd like to ask you questions about the nature of the exam:<br>- What format does the exam take? Is it written or oral?<br>- Are you generally more afraid of written/oral (repeat what was said) exams, compared to exams that have a different format?<br>- Is it a repetition exam?<br>- *If not yet clear:* Does the test anxiety also have to do with the subject in which the exam takes place? In which subject is the exam? What does this subject involve?<br>- What makes the exam so difficult or scary for you?<br>- How often are you afraid of an exam to this extent or are you stressed because of the exam (in every learning phase or especially now)? (Possibly why especially now?)

used for the treatment of heat-induced pain. And there too we found large placebo effects. A positive placebo effect has also been shown for test anxiety. This has been shown recently by a study from Germany, where they tested open placebos also in students.<br>We are now considering the possibility that if placebos work, even though we know that they are placebos, then we could simply omit the sugar pill and imagine the pill and still have all the placebo effects. A reaction to placebos is not only triggered by the placebo pill itself, but also by the imaginative meaning that is both consciously and automatically attributed to the placebo pill. Imagination research shows, for example, that the idea of something activates the same areas of the brain as when one actually sees or experiences something. A study has also shown that the idea of exercising in a gym can already lead to muscle growth. Accordingly, it is possible to imagine taking this pill and achieve a similar effect as if you were taking a real pill. And this is exactly what I would like to discuss and practice with you. Okay for you?	study from Germany, where they tested open placebos also in students.	
**3. Constructing a personally meaningful pill**<br>The first step is to find an imaginary pill for you. Recall the positive state, that you described earlier and the experience of relief you would like to feel. Suppose there was a pill that could bring you to that state, what effects would that pill have, how would it help you reach that state? Imagine there was a pill that could have all these positive effects. What would this pill look like (regarding color, shape and size)? And is the pill packaged also?	**3. One mechanism of placebos: Conditioning**<br>Next, I would like to explain in more detail why placebos can alleviate symptoms. A very important explanation is that the body automatically reacts to the intake of medication. From an early age we learn that pills and effects are related, it results in a learning effect, so to speak. Accordingly, swallowing the pill alone can lead to symptom relief. The physiological reaction of our body to placebos is comparable to this. We	**3. Talking about the problem (test anxiety) and the wished-for state**<br>- Can you tell me specifically about a bad exam (it can also be a lecture or something similar) that you have had in the past and where you were very afraid? [Ask person to actually name an exam, the more specific the better]. I ask you now to think back to that bad exam/situation [wait until person remembers]. Now, using that previous exam

[Wait and trust, that the person will come up with a pill. If a picture of such a pill is cannot be formed, then offer pill characteristics to choose from, for example: "The pill could be round, oval, (...)" etc.]	know that when placebos work, they release neurotransmitters such as endorphins and dopamine, automatically activating specific areas of the brain. These neurotransmitters, in turn, can relieve symptoms or have a positive effect on mood.	situation, can you describe to me what the symptoms of your exam anxiety felt like? What were the sensations in your body based on your experience? What are thoughts and emotions that went through your mind?<br>- Could you use a previous exam situation to describe what the symptoms feel like? I ask you now to think back to that bad exam/situation. Can you now describe to me, based on this previous exam situation, what the symptoms of your exam anxiety felt like? What are the sensations in your body? What are your thoughts and emotions when you experience this anxiety? Now, thinking of the upcoming exam, if you had to specify how strong these symptoms are from 0-10 (not at all – very strong) in this moment, what would you say?<br>- And now can you describe how you would like to feel in your exam phase/during the exam?
**4. Taking the IP**<br>Now imagine the pill described in detail as if it were a real pill. You can ascribe so much reality to the pill that taking it is experienced as if you were swallowing a real pill. It may take some practice. The effect may be stronger and the procedure easier for you if you have done it several times. Now I would suggest that you take your imaginary pill, to try this. You can close your eyes, if you want to. Just think of it as a regular pill. Imagine the pill and how it is packaged. Imagine how you take the pill out of the packaging and how you hold it in your hand. Bring it to your mouth.	**4. An open attitude towards the treatment can be helpful but is not necessary**<br>It's also absolutely okay if you have doubts that placebos work. As mentioned before, placebos can work automatically, which means they can work even if you have doubts.	**4. Learning strategies**<br>- Now I'm still wondering what your general learning strategies are: Do you work in study groups or more alone or both?<br>- Do you study with summaries, mind maps, study plans or flashcards?<br>Thank you very much for your answers to the many questions, it is very informative.

Swallow the pill slowly. Now it is in your body and starts to work. Maybe you can already feel the effects of the pill. Try to feel what the pill does to you. Maybe the pill has also other effects, such as making your mouth dry. You might get warm or a little dizzy.<br>You have now had your first experience of such an imaginary pill taking. Try to remember this state so that you can recall it on your own.<br>Now, if you had to indicate again after taking your imaginary pill how strong at the moment your symptoms are from 0-10 when you think about the upcoming exam, what would you say?		
**5. Suggestions for self-administering in real life and building adherence**<br>For the effect of this intervention, it is now important that you take such an imaginary pill twice a day from the time when you receive a reminder per e-mail: once in the morning and once in the evening, in order to reach the desired state (up to the exam). Before taking the pill, take a little time to recall the image of the pill you have just described.<br>I also ask you to fill out the announced surveys once a week until the exam. You will receive an e-mail with the link at the right time, so that you remember to do it.<br>Then one more thing: In order for us to really be able to identify what the effects of an <u>imaginary</u> pill are, we ask you not to take sweets like Sugus or Tiktak to make it easier for you to imagine. As said before, it is best to simply take your time and take the imaginary pill twice a day for three weeks.	**5. Taking the pill faithfully is important**<br>Therefore, it is important that you take the placebos regularly and according to the prescription. This means for you that you have to take the placebo pills faithfully in order to feel an effect. It is important for you to know that for some people the effects occur earlier and for others later. When you take the pills, we recommend that you also be aware of what the pills are supposed to help you against, i.e., to achieve the positive state you described earlier.<br>I am aware that this may sound unfamiliar to you at first. However, we want to find out what happens to your symptoms when you take placebo pills every day. Therefore, I would like to encourage you to give the open placebo treatment a chance and see what happens.<br>Now you may open the parcel and take out the box with the placebo pills in it. Please take two pills every day for the next three weeks (until the exam) from the time you receive a reminder by e-mail. It	

We will also send you daily reminders that you are reminded to take the imaginary pill.<br>I am aware that the concept of the imaginary pill may sound strange to you at first. But we would like to find out whether you can reach the desired state if you imagine taking a pill every day to relieve your test anxiety. We have developed this procedure here at the university in collaboration with experts from all over the world and we really believe in the effectiveness of this treatment. Also, because there are already several cases from the clinic where the imaginary pill treatment has shown very good effectiveness. Therefore, I would like to encourage you to give the imaginary pill a chance and see what happens.	is best to take the pills at the same time in the morning and in the evening. There is also a small envelope in the package, which you can open right away. On the envelope you will find information on how to take the pills. As we said, we will send you daily reminders to remember to take your pills. If you take two pills a day for three weeks, that's a total of 42 pills. There are 50 pills in the package, which means there are 8 pills too many. You don't have to send them back to us (you can take them at a later date, for example). During the three weeks please take always two pills per day and not more or less.<br>I also ask you to fill out the announced surveys once a week until the exam. You will receive an e-mail with the link at the right time to make sure you remember to do this.	

Open-ended questions

Open-ended questions in open-label placebo group

1. What do you think about the idea of taking placebo pills? (open-ended question)
2. Do you find the placebo pill has generally helped you to be less anxious/stressed before the exam? Yes/No
3. For which symptoms did the placebo pill help to which extent 0% (the pill did not help at all) - 100% (the pill helped 100%)
   - Concerning excitement (emotional and physical tension)
   - Concern (thoughts about failure, self-doubt)
   - Regarding distraction (distraction from the task by irrelevant thoughts)
   - Regarding confidence (self-worth)
4. Did you assume that the placebo pills would work or were you skeptical? (open-ended question)
5. What do you think was in the placebo pills ? (open-ended question)
6. What did you learn by participating in this treatment study ? (open-ended question)
7. Do you have any other comments ? (open-ended question)

Open-ended questions in imaginary pill group

1. In general, how open are you to taking a pharmacological pill for your test anxiety ? 0% (not at all open) to 100% (very open) (slider).
2. Do you find the imaginary pill helped you to be less anxious/stressed before the exam ? Yes/No
3. For which symptoms did the imaginary pill help to what extent 0% (the pill did not help at all) - 100% (the pill helped 100%)
   - Regarding excitement (emotional and physical tension)
   - Regarding concerns (thoughts about failure, self-doubt)
   - Regarding distraction (distraction from the task by irrelevant thoughts)
   - Regarding confidence (self-worth)
4. How difficult was it for you to imagine the imaginary pill? 1 (very easy) - 7 (very difficult)
5. How well could you imagine the following aspects of the imaginary pill (0 - not at all well to 100 almost identical to a real pill):
   - Seeing the pill (visualization)
   - Tasting the pill
   - Feeling the pill
   - Effects of the pill
6. Did you find it easier to visualize and take the imaginary pill during the study?
   - Yes, it was easier
   - It did not change
   - No, it became more difficult

8. What do you think about the idea of taking an imaginary pill ? (open-ended question)
9. Did you assume that the imaginary pill would work or were you skeptical ? (open-ended question)
10. Did you learn anything from participating in this treatment study? If yes, what? (open-ended question)
11. Do you have any other comment? (open-ended question)

Open-ended questions in control group

1. Were you disappointed that you were in the control group? Yes/No
2. Is there anything else you would like to comment on? (open-ended question)

Supplementary Table

Table S1

Mean values for subscales of the test anxiety questionnaire for all assessed timepoints.

		T1	T2	T3	T4	T5
	Group (n)	*M (SD)*				
worry	IP (55)	13.27 (3.36)	12.36 (3.65)	12.18 (3.58)	12.36 (3.99)	10.42 (10.18)
	OLP (59)	14.42 (2.96)	13.85 (3.00)	13.00 (3.44)	13.66 (3.47)	10.86 (10.60)
	CG (59)	13.95 (3.50)	14.05 (2.75)	13.89 (3.48)	14.14 (3.65)	11.49 (9.97)
emotionality	IP (55)	11.00 (3.25)	9.69 (2.81)	9.24 (2.84)	9.80 (3.13)	9.58 (3.47)
	OLP (59)	12.08 (3.52)	10.97 (2.78)	10.07 (2.83)	10.24 (3.15)	10.14 (3.33)
	CG (59)	11.25 (2.89)	11.83 (3.39)	11.75 (2.88)	12.10 (3.84)	11.93 (3.99)
interference	IP (55)	10.42 (3.26)	10.29 (3.11)	9.36 (3.25)	9.38 (3.31)	6.85 (2.38)
	OLP (59)	11.34 (3.14)	10.66 (2.82)	9.95 (3.13)	10.20 (3.14)	7.20 (2.72)
	CG (59)	11.31 (3.14)	11.98 (3.17)	11.59 (3.40)	11.69 (3.27)	8.34 (2.91)
lack of confidence	IP (55)	14.16 (2.94)	12.91 (2.71)	13.05 (2.65)	13.15 (3.05)	13.00 (3.49)
	OLP (59)	14.51 (2.47)	13.83 (2.64)	13.68 (2.68)	13.47 (2.93)	13.44 (3.53)
	CG (59)	14.15 (2.32)	14.22 (2.09)	14.24 (2.74)	14.17 (2.83)	14.02 (2.96)

Note. *ASS-SYM* Änderungssensitive Symptomliste (general well-being), *CG* control group, *IP* imaginary pill, *M* mean, *OLP* open-label placebo, *PAF* Prüfungsangstfragebogen (test anxiety questionnaire), *PSQI* pittsburgh sleep quality index, *SD* standard deviation.
